# Experimental Investigations and Constitutive Modeling of the Dynamic Recrystallization Behavior of a Novel GH4720Li Superalloys with Yttrium Micro-Alloying

**DOI:** 10.3390/ma17153840

**Published:** 2024-08-02

**Authors:** Zehua Yan, Jiahui Hu, Shouxue Sun

**Affiliations:** Rongcheng College, Harbin University of Science and Technology, Weihai 264300, China; jiahuihu2003@163.com (J.H.); ssxue0825@163.com (S.S.)

**Keywords:** novel GH4720Li superalloy, microstructure, hot deformation behavior, constitutive model, DRX model

## Abstract

GH4720Li is an advanced nickel-based alloy celebrated for its remarkable high-temperature strength. This study aimed to investigate the dynamic recrystallization (DRX) behavior of novel GH4720Li superalloys microalloyed with 0.3Y via hot compression tests. A constitutive model was formulated to simulate the DRX behavior. Utilizing the stress–strain curve, the activation energy for the alloy was determined using both the Arrhenius model and the Z-parameter equation, resulting in 1117.916 kJ/mol. The microstructure evolution analysis conducted revealed that lower strain rates at elevated temperatures effectively hindered the occurrence of DRX. Conversely, the increase in the strain rate promoted DRX, producing uniform, equiaxial grains. Recrystallization calculations, along with validation experiments, demonstrated the efficacy of the Avrami model in establishing a DRX model for the alloy during hot deformation. This model accurately quantified DRX percentages under varying deformation parameters, showcasing strong agreement with the microstructure test results. The predictive capability afforded by the developed models offers valuable insights for optimizing the alloy’s forging process. During the compression of the novel GH4720Li superalloy, DRX initiates when the dislocation density in a specific region surpasses a critical threshold. Concurrently, dislocation accumulation near the grain boundaries exceeds that within the grains themselves, highlighting that newly formed DRXed grains primarily emerge along the deformed grain boundaries.

## 1. Introduction

Superalloy materials play a crucial in advanced aero-engines, primarily aimed at enhancing mechanical properties and high-temperature resistance [1,2,3]. Among the crucial components under extensive research and development are aero-engine discs and blades. GH4169 alloy, prominently featured in the latest generation of gas turbine engines, boasts a maximum service temperature of 650 °C [4,5]. Nevertheless, its temperature capabilities are constrained by the dissolution temperature of the substable γ″ phase [6]. On the other hand, GH4720Li, an advanced nickel-based alloy characterized by a high content of γ″, surpasses GH4169 in terms of stability and service temperature [7]. Particularly, the complete dissolution temperature of the primary reinforcing phase γ’ in GH4720Li alloy exceeds 1150 °C [8]. Although GH4720Li alloy is utilized in turbine discs and blades, its high γ’ phase content presents challenges, impacting hot working performance and microstructure control [9]. Nevertheless, GH4720Li alloy continues to be a focal point of research worldwide due to its exceptional high-temperature mechanical properties [10,11,12]. Forging exhibits potential in improving the internal microstructure and mechanical properties of GH4720Li superalloy, providing high productivity and versatility [13]. Therefore, forging stands as the primary processing method for GH4720Li superalloy. However, the alloy’s extensive alloying poses challenges, such as substantial resistance to deformation and a narrow suitable heat processing range, which complicates large deformation processes [14].

For various newly developed metallic materials, hot processing is essential in controlling the microstructure, thereby influencing the properties of the final product [15,16]. In materials with high stacking fault energy, dynamic recovery (DRV) prevails as a softening process, whereas those with low to mid-level fault energies are governed by DRX [17]. However, traditional approaches that rely on trial-and-error methods for understanding hot deformation behavior have limitations, such as poor timeliness and high costs. To overcome these limitations, physical simulation has emerged as a crucial alternative. It allows for the simulation of real working conditions by controlling the parameters [18]. Recently, hot compression simulation has been widely applied to various materials, including alloys [19,20] and composites [21,22]. Various quantitative models have been proposed to elucidate different aspects of the behavior of Ni-based superalloys during high-temperature deformation [23,24,25]. These include the constitutive model for stress–strain relationship, the processing map model for hot processing performance, and the recrystallization model focusing on DRXed grain nucleation [26,27,28]. Achieving a thorough understanding of the DRX in Ni-based superalloys is essential for enhancing their comprehensive performance and achieving a uniform, fine tissue structure. It is crucial to recognize that hot deformation parameters significantly influence the microstructural evolution of these superalloys. Contemporary research on hot deformation behavior emphasizes high-temperature deformation analysis, utilizing hot processing maps to guide industrial production and exploring DRX behavior [29,30,31]. Researchers have made notable contributions to this field by developing constitutive, intrinsic, and kinetic models that take into account critical factors such as Zener–Hollomon parameters, DRX critical models, and activation energies [32,33,34]. These models accurately forecast flow stress and DRX behavior, demonstrating good alignment with experimental data and microstructural analyses. Moreover, information criteria-based models, finite element simulations, and hot compression tests have further enriched our comprehension. In conclusion, collaborative research endeavors have substantially progressed the understanding of hot deformation behavior, dislocation sub-structure, and microstructure regulation in Ni-based superalloys, thereby facilitating precise microstructure control in their advancement.

Renowned for its chemical activity, the rare earth element Y finds applications across diverse fields, including metallurgy, chemistry, and surface engineering [35]. Even in trace amounts, Y substantially enhances oxidation resistance, creep resistance, and longevity in alloys, rendering it highly promising [36]. Given the ongoing quest for higher aero-engine thrust-to-weight ratios, there is a mounting need for key components endowed with enhanced high-temperature resistance and strength. Consequently, there is an increasing adoption of new superalloys incorporating the rare earth element Y [37,38,39]. In line with this trend, this study focuses on the novel GH4720Li superalloys with yttrium micro-alloying. By employing principles such as hot deformation behavior, work hardening, and DRX, researchers calculate the proportion of DRX. The study establishes a DRX model for the alloy’s hot deformation, providing quantitative predictions. The accuracy of this model is validated through microstructure tests, providing theoretical foundations and experimental data crucial for formulating the forging and open billet process of the novel GH4720Li alloys with yttrium micro-alloying. This, in turn, supports subsequent forging processing and the realization of the desired microstructure through forging. During production and processing, selecting appropriate plastic processing methods based on equipment conditions, actual needs, and performance requirements can achieve the best quality of formed parts at the lowest cost.

## 2. Materials and Methods

**Figure 1:**
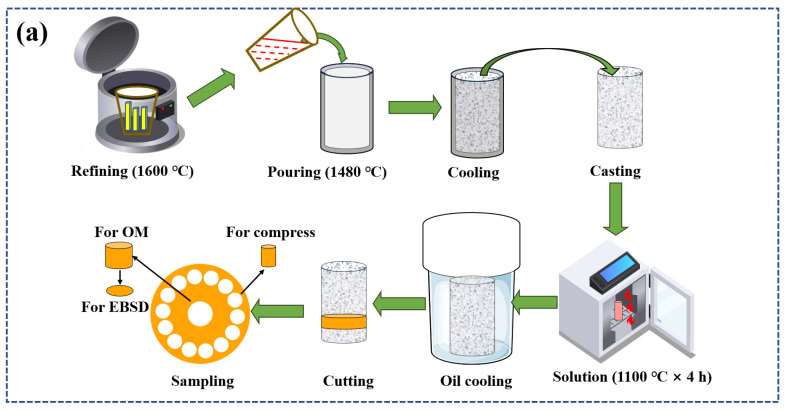

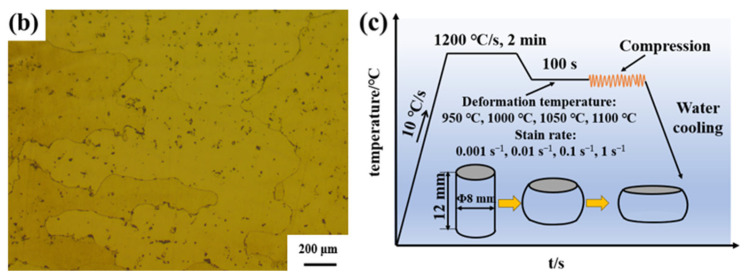
Flowchart, OM image, schematic figure of the compression experiment specimen: (**a**) flowchart, (**b**) OM image, and (**c**) schematic figure.

The novel GH4720Li alloys with 99.9% yttrium micro-alloying were produced by remelting and smelting, with their composition shown in Table 1. Alloy ingots were fabricated using vacuum arc remelting furnaces (YC2016-0022, Linyi Research and Innovative Materials Technology Co., Ltd., Linyi, China) and induction melting furnaces (YC2018-0022, Linyi Research and Innovative Materials Technology Co., Ltd., Linyi, China), and the chamber was evacuated to 3 × 10^−3^ Pa using high-purity argon. Refining was carried out at 1600 °C lasting 10 min, pouring the molten alloy at 1480 °C into a 120 mm diameter Al_2_O_3_ ceramic mold. Post casting, the sample underwent a heat treatment at 1100 °C for 4 h, and then oil-cooled. Compressed specimens were bisected using WEDM (JA800, Jiangsu Sevis CNC Technology Co., Ltd, Suzhou, China) with the following parameters: a 24 μs pulse width, an 8 μm discharge gap, a 37.82 mm^2^/min wire speed, and a 0.1428 duty cycle. See Figure 1a for the material preparation flowchart. To calibrate the Gleeble-3800, thermocouples were connected, and the machine was powered on to access the calibration menu. Zero calibration was carried out according to on-screen instructions, setting the baseline readings. Span calibration involved applying known temperatures to the thermocouples. Prior to compression, lubricant and a 0.1 mm tantalum sheet were placed to reduce friction and ensure a uniform heat distribution. Hot compression specimens (Φ8 mm × 12 mm) were tested at 950–1100 °C, strain rates 0.001–1 s^−1^, and a true strain of 0.7. During the test, the material was heated to 1200 °C at 10 °C/s and held for 120 s for uniform heating. The material was then cooled to the experimental temperature at 5 °C/s and held for 100 s to eliminate gradients. After compression, water quenching preserved the microstructure, and the data were automatically collected. After cutting, the sample was sequentially sanded with 600#, 1000#, and 2000# grit sandpaper. The sample was then polished with aluminum oxide until a mirror-like finish was achieved. The samples were then etched with Kalling’s solution (100 mL HCl + 100 mL anhydrous ethanol + 5 g CuCl_2_) and analyzed using an optical microscope (OM: GX71, Olympus, Shinjuku, Japan) at 400× and 1000× magnifications. Etching was carried out by dispensing a corrosive solution onto the polished surface. Etching involved adding a drop of the corrosive solution every 10 s for 50 s. An OM image of the solution-treated alloy is shown in Figure 1b. Electrolytic etching used a 9:1 C_2_H_5_OH and HClO_4_ solution, applying 20 V for 25 s while at approximately 0 °C. Post etching, the sample was cleaned with ethanol and dried with a hair dryer. Electron backscatter diffraction (EBSD: JSM-7800F, Oxfordshire, UK) images were captured at 20 kV and 100× magnification, with a 0.5 μm step and a 500 μm × 500 μm field. The EBSD images were processed and analyzed using Channel 5 software. The experimental specimens and procedure are illustrated in Figure 1c.

## 3. Results and Discussion

### 3.1. Rheological Behavior and Microstructural Evolution of the Novel GH4720Li Superalloys

#### 3.1.1. Rheological Behavior of Alloys

**Figure 2:**
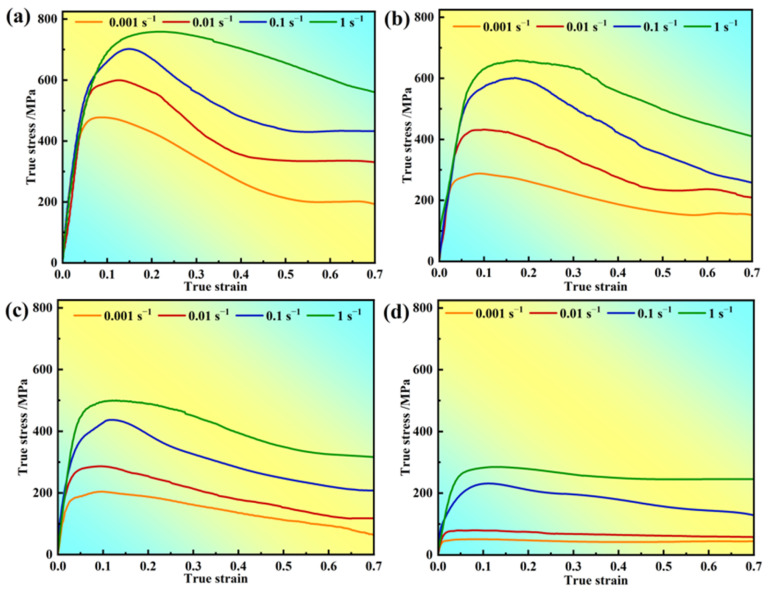
Stress–strain curves of the novel GH4720Li superalloy: (**a**) 950 °C, (**b**) 1000 °C, (**c**) 1050 °C, and (**d**) 1100 °C.

Figure 2 presents the stress–strain curves of the novel solutioned GH4720Li alloy under various deformation conditions, highlighting the significant impact of the deformation parameters on rheological stress. True stresses increase because of reduced grain boundary (GB) migration at low temperatures and shorter deformation times at high strain rates. Additionally, all of the rheological stress profiles under the investigated deformation conditions exhibit distinct work hardening and dynamic softening phenomena. During the initial deformation stage, slip system activation rapidly increases dislocation density, causing peak stress and a sudden rise in true stress. As dislocation density increases, DRV and DRX occur at a critical density. Subsequently, dislocations are annihilated during DRXed grain growth, leading to a gradual decrease in true stress [40]. Upon reaching a critical true strain, work hardening and recrystallization softening reach a dynamic balance, manifesting as steady-state rheological stresses. Furthermore, under constant strain rate conditions, true stress significantly decreases with increasing deformation temperature due to enhanced GB mobility and weakened γ’ phase pinning [41]. Conversely, as the strain rate increases, γ’ phase dissolution weakens, and rapid dislocation proliferation and strong plugging result in a significant stress increase [42]. Figure 2 also illustrates that increasing temperature significantly reduces the material’s flow stress levels, with the peak stress at 950 °C being three to four times lower than at 1100 °C. Several factors contribute to this phenomenon, primarily stemming from two aspects. Firstly, higher deformation temperatures enhance thermal activation, leading to an increase in the rate of DRXed nuclei formation controlled by thermal activation. Additionally, elevated temperature also facilitates GB migration and DRXed grain growth, thereby strengthening the dynamic softening effect. This offsets or surpasses work hardening, reducing flow stress. Higher temperatures increase atomic kinetic energy, enhancing atomic motion. This process weakens the bonding forces between atoms, consequently reducing the material’s critical shear stress. As a result, the occurrence of multiple slip systems in the material is promoted, leading to a decrease in the material’s deformation resistance.

#### 3.1.2. Influence of Deformation Parameters on Flow Stresses in Alloys 

Numerous studies have shown that the material’s rheology is controlled by thermal activation. The flow stress–strain relationship can be described by power, exponential, and hyperbolic sine functions [43], as expressed in Equation (1).

(1) $$\left\{\begin{array}{l} \dot{\varepsilon }=A_{1}\sigma^{n1}\exp\left(-\frac{Q}{RT}\right)\\ \dot{\varepsilon }=A_{2}\exp\left(n_{2}\sigma \right)\exp\left(-\frac{Q}{RT}\right)\\ \dot{\varepsilon }=A{\left[\sin(\alpha \sigma )\right]}^{n}\exp\left(-\frac{Q}{RT}\right) \end{array}\right.$$

where $\dot{\varepsilon }$ is the strain rate (s^−1^), *σ* is the flow stress (MPa), *n* is the stress index, *Q* is the heat activation energy (kJ/mol), *T* is the heat deformation temperature (K), and *A*_1_, *A*_2_, *A*, *β*, *α*, *n*_1_ are material constants. Deriving Equation (1) yields Equation (2).

(2) $$\left\{\begin{array}{l} \ln\dot{\varepsilon }=\ln A_{1}+n_{1}\ln\sigma -\frac{Q}{RT}\\ \ln\dot{\varepsilon }=\ln A_{2}+\beta \sigma -\frac{Q}{RT}\\ \ln\dot{\varepsilon }=\ln A+n\sinh(\alpha \sigma )-\frac{Q}{RT} \end{array}\right.$$

**Figure 3:**
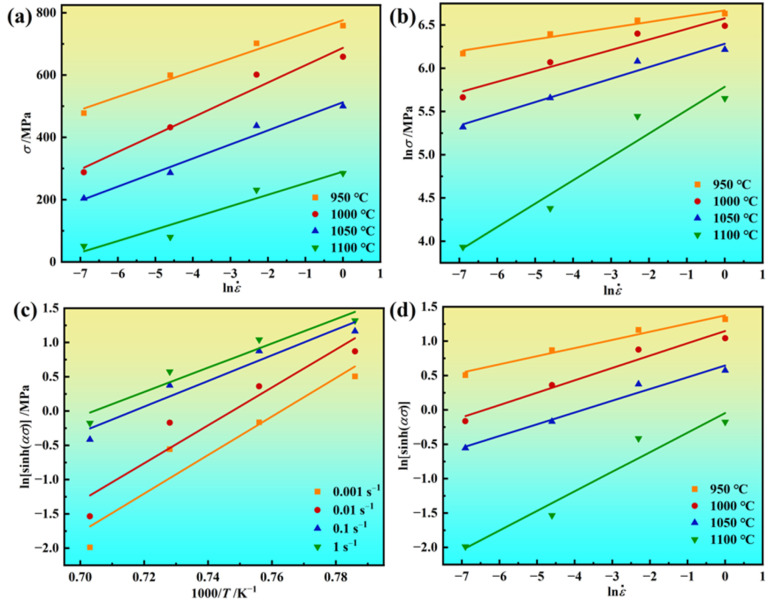
Relationship among *σ*, $\dot{\varepsilon }$, and *T* of the novel GH4720Li superalloy ingot under hot deformation: (**a**) *σ* − ln$\dot{\varepsilon }$, (**b**) ln*σ* − ln$\dot{\varepsilon }$, (**c**) lnsinh(α*σ*) − *T*, and (**d**) lnsinh(α*σ*) − ln$\dot{\varepsilon }$.

From Equation (2), the *β*-value and *n*_1_-value are determined from the relationships between *σ* and $\ln\dot{\varepsilon }$, ln*σ*, and $\ln\dot{\varepsilon }$, as illustrated in Figure 3a,b. By conducting linear regression analysis on the relationships between $\dot{\varepsilon }$ and *σ* at different deformation temperatures, there exists a satisfactory linear relationship. Based on the determination of *α* = *β*/*n*_1_, the *α*-value was established. By examining the relationship between lnsinh(*ασ*) and ln*ε*, as illustrated in Figure 3c, the *n*-values under different temperature conditions were obtained. It can be observed that the linear correlation coefficients of the regression equation are all higher than 90%, and analysis shows that the *n*-value gradually increases with rising temperature. Additionally, the highest *n*-value does not exceed 9, while the lowest value remains no less than 3 across all temperature ranges. Studies have indicated that the stress exponent is associated with the deformation mechanism [44]. An *n*-value close to 3 signifies that deformation is primarily controlled by dislocation creep. An *n*-value above 5 indicates the dominant mechanism is high-temperature creep caused by lattice self-diffusion. When deforming, the novel GH4720Li superalloy consistently exhibits high *n*-value, indicating that its deformation is primarily governed by high-temperature creep caused by lattice self-diffusion. Figure 3d shows that the stress of the novel GH4720Li superalloy increases with decreasing temperature, exhibiting a strong linear relationship (not less than 0.9), suggesting that Equation (2) can better depict the relationship between *σ* and $\dot{\varepsilon }$. When the novel GH4720Li superalloy ingots are deformed at temperatures of 950 °C, 1000 °C, and 1050 °C, the alloy exhibits high stress index values (ranging from 5.57 to 8.41). This suggests its deformation is predominantly governed by high-temperature climbing caused by lattice self-diffusion. Conversely, when the alloy undergoes deformation at 1100 °C, the maximum *n*-value is 3.51. This indicates its deformation process is controlled by the viscous slip of dislocations.

#### 3.1.3. Effect of Strain Rate and Temperature on Alloy Microstructure

**Figure 4:**
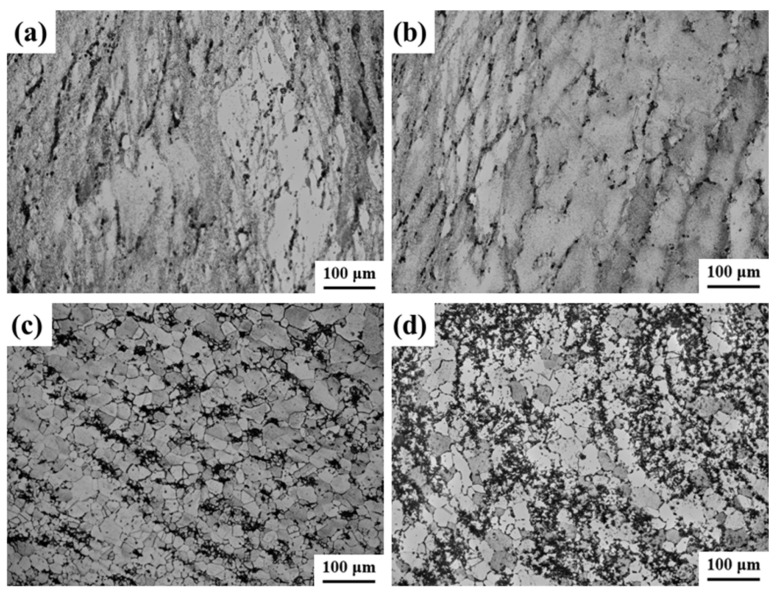
OM images of compressed alloy with different temperatures: (**a**) 950 °C, (**b**) 1000 °C, (**c**) 1050 °C, and (**d**) 1100 °C.

Figure 4 illustrates the novel GH4720Li superalloy microstructure at different temperatures. At 950 °C, only a limited degree of DRXed grains can be observed, with numerous elongated original grains retained post hot deformation. The low deformation temperature impedes GB migration, hindering the growth of grains undergoing DRX, as shown in Figure 4a. At 1000 °C, the alloy undergoes evident DRX, forming small equiaxed grains within the original grain boundaries, creating a distinctive necklace-like structure, as depicted in Figure 4b. At 1050 °C, the percentage of DRXed grains further increases compared to 1000 °C. The higher temperature allows the newly formed fine equiaxed grains to expand into the original coarse grains, fostering DRXed grain growth, as illustrated in Figure 4c. At 1100 °C, the percentage of DRX continues to rise, approaching 100%. This phenomenon is primarily attributed to the favorable conditions at higher deformation temperatures. Enhancing GB migration, increased DRX nucleation, and DRX development. At the specified deformation temperature, fine equiaxed grains formed via DRX are evident, as illustrated in Figure 4d. These grains grow by assimilating the surrounding original coarse grain deformations, ultimately resulting in the presence of individual grains. This observed phenomenon is closely associated with the thermally activated nature of DRX, which typically initiates near grain or subgranular boundaries. As the deformation temperature increases, the heightened thermal motion weakens interatomic bonding forces, facilitating more extensive diffusion. Compared to lower temperatures, there is a heightened likelihood of dislocation climbing, slipping, and cross-slip shifts. Simultaneously, this enhanced the capacity of GB migration, increased DRX nucleation, and fostered the occurrence of DRX behavior. However, higher temperatures cause the growth rate of newly formed fine equiaxed grains via DRX to surpass the nucleation rate. Consequently, these grains coarsen by assimilating the surrounding deformed grains.

**Figure 5:**
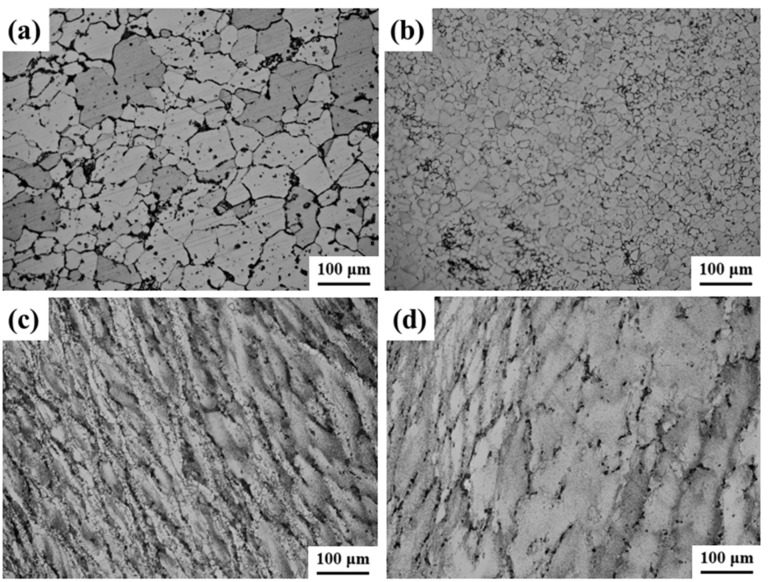
OM images of compressed alloy with different strain rates: (**a**) 0.001 s^−1^, (**b**) 0.01 s^−1^, (**c**) 0.1 s^−1^, and (**d**) 1 s^−1^.

Figure 5 illustrates the microstructure of the novel GH4720Li superalloy under various strain rates. At a strain rate of 0.001 s^−1^, the original coarse grains are predominantly transformed into refined grains. The low strain rate allows small equiaxed grains to form gradually at high temperatures, assimilating surrounding grains and coarsening, as shown in Figure 5a. As the strain rate increases to 0.01 s^−1^, complete DRX occurs. Newly formed fine equiaxed grains coarsen, but not as severely as at 0.001 s^−1^, as shown in Figure 5b. Further increasing the strain rate to 0.1 s^−1^, the proportion of DRX decreases, leading to the formation of distinct necklace-like grains. Additionally, owing to the elevated deformation temperature, these newly formed DRXed grains exhibit increased size. Furthermore, a significant number of original deformed grains, elongated perpendicular to the compression direction, are retained, as depicted in Figure 5c. Upon reaching a strain rate of 1 s^−1^, the proportion of DRX is further diminished compared to 0.1 s^−1^. Because of the shortened compression time, the newly formed DRXed grains at the grain boundaries do not have sufficient time to grow, as illustrated in Figure 5d. Overall, the percentage of DRXed grains exhibits a gradual decrease with increasing strain rate. At lower strain rates, characterized by prolonged deformation times, the grains undergo an extended duration at high temperatures, facilitating atomic movement and GB migration. Consequently, the DRX nucleation rate increases, leading to a higher percentage of DRXed grains. Conversely, at higher strain rates, where deformation times are shorter, the DRXed grains experience a brief duration at high temperatures. This leads to nucleation occurring primarily from original coarse grains distant from the grain boundaries, resulting in a lower DRX percentage. Furthermore, the DRXed grain sizes diminish with increasing strain rate, promoting grain refinement and accumulating deformation energy at defects. With a further increasing strain rate, the deformation time shortens progressively. The pinning effect of solute atoms on dislocations impedes atomic movement and GB migration. Thus, the DRXed grains have less time at high temperatures, hindering significant growth.

### 3.2. Analysis of the Intrinsic Behavior of Alloys

Generally, the deformation behavior of superalloys follows thermal activation principles, typically described by an Arrhenius-type intrinsic relationship model [45]. In this model, the Z-parameter (Zener–Hollomon parameter) is expressed in Equation (3).

(3) $$Z=\overset{\cdot }{\varepsilon }\exp(\frac{Q}{RT})=f\left(\bar{\sigma }\right)=\left\{\begin{array}{l} A{[\sin h(a\bar{\sigma })]}^{n}\\ A_{1}{\bar{\sigma }}^{n_{1}}\\ A_{2}\exp(\beta \bar{\sigma }) \end{array}\right.$$

where *Q* represents the hot deformation activation energy (kJ/mol), *R* is the gas constant (8.314 kJ/mol·K), *A*, *A*_1_, *A*_2_, *n*_1_, *α*, *β* are material constants, and *n* is the stress exponent. In this study, the hydrodynamic behavior of the material is characterized using the stress state corresponding to the peak strain condition, obtained by taking the natural logarithm of Equation (4).

(4) $$\ln\overset{\cdot }{\varepsilon }+\frac{Q}{R}(\frac{1}{T})=\left\{\begin{array}{l} \ln A+n\ln[\sin h(a\bar{\sigma })]\\ \ln A_{1}+n_{1}\ln\bar{\sigma }\\ \ln A_{2}+\beta \bar{\sigma } \end{array}\right.$$

where *β*, *n*_1_, and *n* can be obtained by taking the partial derivatives of Equation (4) at a certain fixed temperature condition, as shown in Equation (5):

(5) $$\left\{\begin{array}{l} \beta ={\left.\left[\frac{\partial \ln\overset{\cdot }{\varepsilon }}{\partial {\bar{\sigma }}_{p}}\right.\right]}_{T}\\ n_{1}={\left.\left[\frac{\partial \ln\overset{\cdot }{\varepsilon }}{\partial \ln{\bar{\sigma }}_{p}}\right.\right]}_{T}\\ n={\left[\frac{\partial \ln\overset{\cdot }{\varepsilon }}{\partial \ln\left[\sin h({\bar{\sigma }}_{p})\right]}\right]}_{T} \end{array}\right.$$

Based on Equation (5), the *β*-value (0.022868) and *n*_1_-value (8.54115) can be calculated using Figure 3a,b. Subsequently, the *α*-value can be determined by the calculation of α = *β*/n_1_ (0.002677 MPa⁻^1^). Additionally, the slope of ln[sinh(ασ)] versus 1/*T* can be derived via linear regression from Figure 3c, yielding a value of 23.03677. Utilizing Figure 3d, the *n*-value can be obtained through linear regression analysis, resulting in a value of 5.8371. Finally, the *Q*-value can be determined using Equation (6), resulting in a value of 1117.966 kJ/mol.

(6) $$Q=R{\left[\frac{\partial \ln\overset{\cdot }{\varepsilon }}{\partial \ln\left[\sin h({\bar{\sigma }}_{p})\right]}\right]}_{T}{\left[\frac{\partial \ln[\sinh(\alpha {\bar{\sigma }}_{p})]}{\partial (1/T)}\right]}_{\dot{\varepsilon }}$$

According to Equation (3), Equation (7) can be obtained.

(7) $$\sinh\left(\alpha \sigma \right)={\left(Z/A\right)}^{1/n}$$

According to Equation (7), Equation (8) can be obtained.

(8) $$\sinh^{-1}\left(\alpha \sigma \right)=\ln\left[\alpha \sigma +{\left(\alpha^{2}\sigma^{2}+1\right)}^{1/2}\right]$$

From Equation (8), the rheological stress of the alloy can be expressed as Equation (9):

(9) $$\sigma =\ln\left\{{\left(\frac{Z}{A}\right)}^{1/n}+{\left[{\left(\frac{Z}{A}\right)}^{2/n}+1\right]}^{1/2}\right\}/\alpha$$

**Figure 6:**
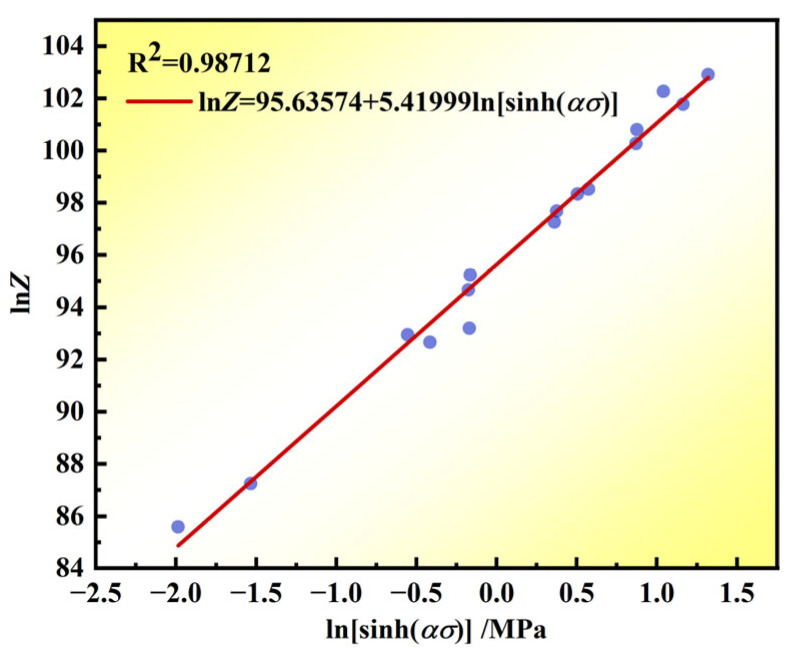
Plot of the linear fit of ln*Z* − ln[sinh(*ασ*)].

Figure 6 shows the linear fit of lnZ and ln[sinh(*ασ*)], yielding a slope value (*n*) of 5.8371 and an intercept value (lnA) of 95.194 (where *A* = 2.202 × 10^41^). Bringing the aforementioned *Q* = 1117966.4 J/mol, *R* = 8.314 J/mol, *n* = 5.8371.

By replacing the aforementioned calculations into Equations (1), (3), and (9), the constitutive equation is acquired as follows:

(10) $$\dot{\varepsilon }=2.202\times 10^{41}{\left[\sinh\left(0.002677\sigma \right)\right]}^{5.8371}\exp\left(1117966.4/RT\right)$$

(11) $$Z=\dot{\varepsilon }\exp\left(1117966.4/RT\right)$$

(12) $$\sigma =\ln\left\{{\left(\frac{Z}{2.202\times 10^{41}}\right)}^{\frac{1}{5.41999}}+{\left[{\left(\frac{Z}{2.202\times 10^{41}}\right)}^{\frac{2}{5.41999}}+1\right]}^{\frac{1}{2}}\right\}/\alpha$$

### 3.3. Critical Strain Model for DRX

#### 3.3.1. Critical Strain Model for DRX

During hot deformation, as the strain increases, numerous dislocations in the alloy become entangled, resulting in work hardening. When strain reaches a certain critical value, sufficient accumulated stored energy initiates DRX. This critical value, known as the DRX critical strain, is a crucial parameter for determining the onset of DRX. An inflection point between work hardening (*θ*) and dynamic softening determines the critical strain, which can be calculated using Equation (13) [46].

(13) θ=dσ/dε=Δσ/Δε

**Figure 7:**
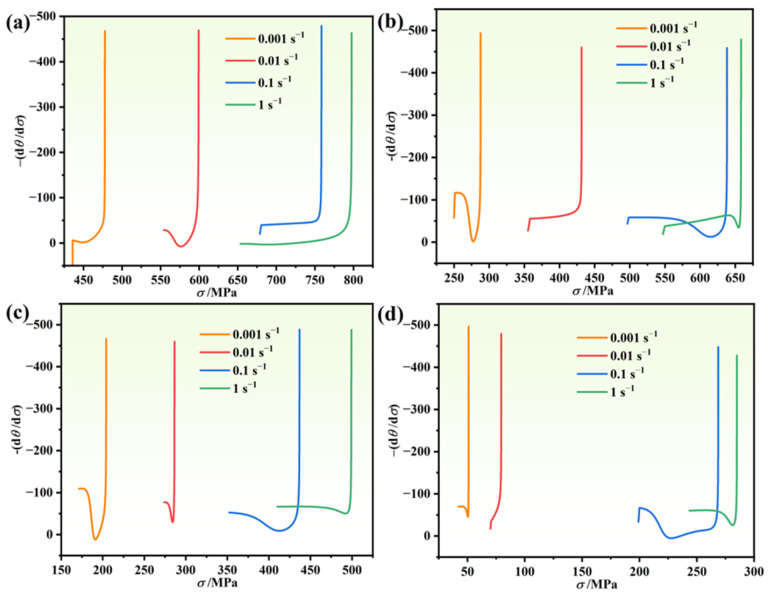
The −(d*θ*/d*σ*) − *σ* curves of Ni-based alloys at different temperatures: (**a**) 950 °C, (**b**) 1000 °C, (**c**) 1050 °C, and (**d**) 1100 °C.

Poliak and Jonas proposed identifying the critical strains as an inflection point in the *θ* − *σ* curve upon reaching the critical state [47]. Thus, analyzing the −*dθ/dσ* − *σ* curve enables the determination of critical conditions for DRX. Figure 7 depicts the −*dθ/dσ* − *σ* curve under various conditions, where the minimum signifies the critical stress. One can see from Figure 7 that at high temperature and low strain rate, the critical strain of the novel GH4720Li superalloy is small, suggesting a higher likelihood of DRX occurrence. Conversely, lower temperatures and higher strain rates result in a relatively large critical strain, indicating a delayed DRX process during hot deformation. This delay stems from the reduced kinetic energy and diffusion ability of atoms at low temperatures, leading to decreased nucleation rates. Additionally, weakened hot vibration of atoms and reduced diffusion rates diminish the efficiency of the dislocation microstructure, inhibiting DRX. As temperature increases, atomic kinetic energy and diffusion rates rise, promoting DRX. Under high strain rates, rapid material deformation results in the proliferation and accumulation of dislocations, intensifying the DRX driving force. However, the short deformation time inhibits dislocation motion, preventing effective stress concentration release and reducing DRX nucleation rates.

During the heat deformation process, the material experiences DRX before reaching peak stress, resulting in a critical strain smaller than the peak strain. Figure 7 illustrates this phenomenon, where the horizontal coordinate at the inflection point indicates the critical stress during DRX. Sellars proposed a critical strain model, expressed as shown in Equation (14) [48].

(14) $$\left\{\begin{array}{l} \varepsilon_{c}=a\varepsilon_{p}\\ \varepsilon_{p}=a_{1}d_{0}^{n_{2}}{\dot{\varepsilon }}^{m_{1}}\exp\left(\frac{Q_{1}}{RT}\right) \end{array}\right.$$

where *ε_c_* and *ε_p_* are the critical strain and peak strain, respectively. *Q*_1_ represents the activation energy of hot deformation, *d*_0_ represents the original grain size, $\dot{\varepsilon }$ represents the strain rate, *R* is the gas constant with a value of 8.314 J/(mol·K), *T* is the deformation temperature, and *a*, *a*_1_, *n*_1_, *m*_1_ are the material-related constants.

**Figure 8:**
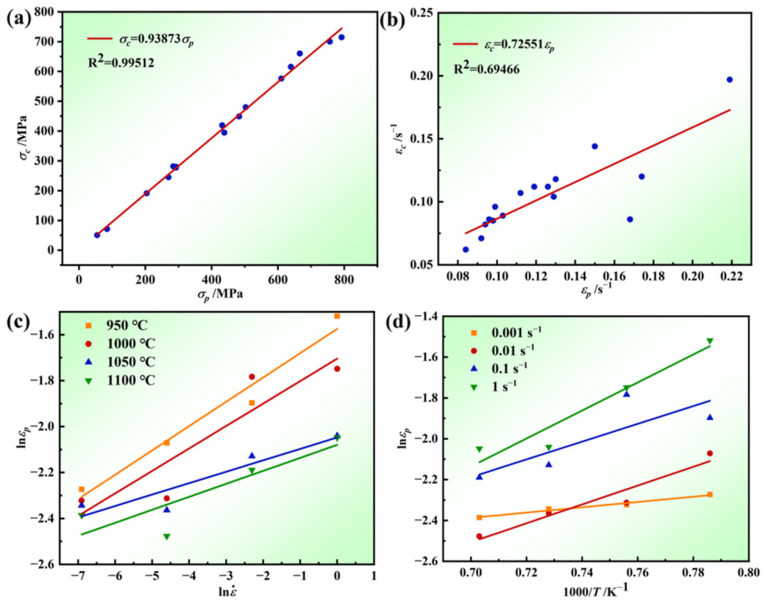
The linear fit of (**a**) *σ_c_* − σ*_p_*, (**b**) ε*_c_* − ε*_p_*, (**c**) $\ln\varepsilon_{p}-\ln\dot{\varepsilon }$, and (**d**) $\ln\varepsilon_{p}-1000/T$.

According to Figure 2, the values of *σ_c_*, *σ_p_*, *ε_c_*, and *ε_p_* for different deformation conditions can be obtained. Figure 8 shows the relationship between *σ_c_* and *σ_p_*, and ε*_c_* and ε*_p_*, and one can see that the two linear fit better; the Sellars model points out that the ratio of *ε_c_* and *ε_p_* is between 0.6 and 0.95, and it can be seen that the conditions are met; therefore, one can obtain the relationship between *σ_c_* and *σ_p_* from Figure 8a:

(15) $$\sigma_{c}=0.93873\sigma_{p}$$

Similarly, one can obtain the relationship between *ε_c_* and *ε_p_* from Figure 8b:

(16) $$\varepsilon_{c}=0.72551\varepsilon_{p}$$

In this study, the alloy undergoes homogenization, making the original grain size’s effect negligible. Therefore, *n*_1_ = 0 is assumed, yielding *a*-value and *a*_1_-value, as obtained using Equation (17).

(17) $$\varepsilon_{p}=a_{1}{\dot{\varepsilon }}^{m_{1}}\exp\left(\frac{Q_{1}}{RT}\right)$$

Taking the logarithms of Equation (17) yields Equation (18):

(18) $$\ln\varepsilon_{p}=\ln a_{1}+\ln{\dot{\varepsilon }}^{m_{1}}+Q_{1}/RT$$

where the values of *m*_1_ and *Q*_1_ can be found by following Equation (19):

(19) $$\left\{\begin{array}{l} m_{1}={\left.\frac{\partial ln\varepsilon_{p}}{\partial ln\dot{\varepsilon }}\right|}_{T}\\ {\left.Q_{1}=R\frac{\partial ln\varepsilon_{p}}{\partial (1/T)}\right|}_{\overset{\cdot }{\varepsilon }} \end{array}\right.$$

As shown in Figure 8c,d, one can obtain *m*_1_ = 0.07016 and *Q*_1_ = 35562.553 J/mol. Subsequently, these values of *Q*_1_ and *m*_1_ are substituted into Equation (18) and averaged to yield *a*_1_ = 0.07127.

Substituting the above data into Equation (14), the critical strain model for the novel GH4720Li superalloy during heat distortion is given, as shown in Equation (20).

(20) $$\left\{\begin{array}{c} \varepsilon_{c}=0.69722\varepsilon_{p}\\ \varepsilon_{p}=0.07127{\dot{\varepsilon }}^{0.07016}\exp(35562.533/RT) \end{array}\right.$$

#### 3.3.2. DRX Volume Fraction Models

The volume fraction of DRX in alloys during hot deformation can be described using the DRX kinetic equation. In this study, the model was formulated using the JMAK equation [49], as shown in Equation (21).

(21) $$\left\{\begin{array}{l} X_{drx}=1-\exp\left[-\beta_{d}{\left(\frac{\varepsilon -\varepsilon_{c}}{\varepsilon_{0.5}}\right)}^{K_{d}}\right]\\ \varepsilon_{0.5}=a_{2}d_{0}^{n_{2}}{\dot{\varepsilon }}^{m_{2}}\exp(\frac{Q_{2}}{RT}) \end{array}\right.$$

where *X*_DRX_ is the volume fraction of DRX, *ε* is the true strain, *ε_c_* is the critical strain of DRX, *ε*_0.5_ is the strain when the volume fraction of DRX reaches 50%, *Q*_2_ is the thermal activation energy of DRX, and *β_d_*, *K_d_*, *a*_2_, and *m*_2_ are constants. In general, the effect of grain size on the DRX volume fraction model is neglected and *n*_2_ = 0. Therefore, the DRX volume fraction model can be simplified to Equation (22):

(22) $$\varepsilon_{0.5}=a_{2}{\dot{\varepsilon }}^{m_{2}}\exp(\frac{Q_{2}}{RT})$$

Equations (21) and (22) indicate that directly calculating Equation (9) is impractical due to the numerous unknowns. Therefore, it becomes essential to obtain the DRX volume integrals, along with determining the values of *β_d_* and *K_d_* in conjunction with the DRX volume parameters. However, obtaining the DRX volume fraction directly and effectively from the microstructure presents a challenge. Moreover, accurately measuring the grain size before and after deformation is highly intricate. Recently, some scholars have developed a computational model to derive the volume fraction of DRX by analyzing the stress–strain curve, in conjunction with Equation (23), enabling the determination of the DRX volume fraction.

(23) $$X_{DRX}=\frac{\sigma_{\mathrm{drv}}-\sigma }{\sigma_{sta}-\sigma_{ss}}$$

where *σ* is rheological stress, *σ_drx_* is restitution stress, *σ_sat_* is the steady state restitution stress, and *σ_ss_* is the steady state rheological stress.

**Figure 9:**
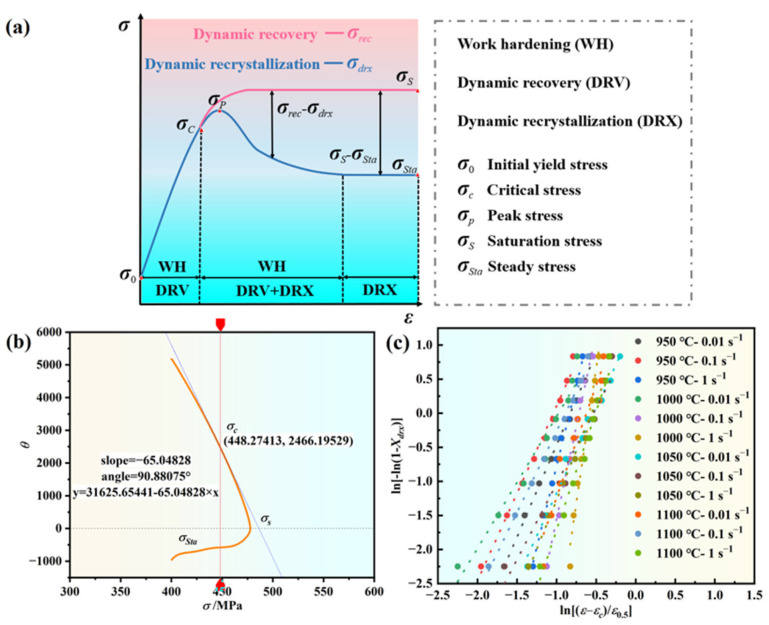
Schematic relationship between dynamic recrystallization kinetics and related curves: (**a**) schematic of DRX and DRV, (**b**) schematic diagram of tangent lines and various stresses during compression of rheological curves, and (**c**) the linear fit of ln[−ln(1 − *X_drx_*)] − ln[(*ε* − *ε_c_*)/*ε*_0.5_.

In Figure 9a, the red curve represents the dynamic recovery (DRV) stress curve, which attains the steady-state return *σ_s_* when the work hardening rate reaches 0. This curve assumes the absence of DRX during heat deformation, with only DRV and work-hardening present. During this stage, the work-hardening rate curve exhibits a linear relationship with the stress value at the critical strain (inflection point) until the work hardening decreases to 0. Figure 9b illustrates the work hardening rate curve using the stress–strain curve of 1100 °C/0.1 s^−1^. The inflection point corresponds to the critical stress (*σ_c_*). The first point of intersection with the zero point of the longitudinal coordinate represents the peak stress (*σ_p_*), while the intersection point of the end of the hardening rate curve with the zero point of the longitudinal coordinate indicates the steady-state rheological stress (*σ_ss_*). The steady-state rheological stress represents the value of the steady-state stress when the alloy material undergoes complete DRX. If the stress–strain curve reaches the steady-state rheological stress value, the end of the work hardening rate curve will intersect with the zero point during the fitting process, enabling a direct reading of the stress value. However, if the curve has not reached the steady-state stage, the end of the work hardening rate curve will not intersect the zero point. Therefore, the stress–strain curve should be partially adjusted downward and fitted to obtain the intersection point with Equation (24), thereby estimating the final steady-state rheological stress value *σ_ss_*.

(24) $$\theta =\frac{d\sigma (\varepsilon )}{d\varepsilon }=k\sigma (\varepsilon )+b$$

where *k* is the slope of the work hardening rate in the DRV stage, and both *k* and *b* are constants.

Taking the logarithms of Equation (21) yields Equation (25).

(25) $$ln\left[-ln\left(1-X_{drx}\right)\right]=ln\beta +kln\frac{\varepsilon -\varepsilon_{c}}{\varepsilon_{p}}$$

From the above equation, it can be seen that $\ln[-\ln(1-X_{drx})]$ is linearly related to $\ln[(\varepsilon -\varepsilon_{c})/\varepsilon_{p}]$. By taking the strain and its corresponding flow stress, the value of $X_{drx}$ is calculated using Equation (25). A linear fitting plot of $\ln[-\ln(1-X_{drx})]$ and $\ln[(\varepsilon -\varepsilon_{c})/\varepsilon_{p}]$ is plotted, as shown in Figure 9c, where *k* was calculated to be 3.19267 and β is 0.5308.

Substituting the *β*-value and *k*-value into Equation (26) yields a model for the volume fraction represented by the Yada model of the alloy.

(26) $$X_{drx}=1-\exp\left[-0.5308{\left(\frac{\varepsilon -0.72551\varepsilon_{p}}{\varepsilon_{0.5}}\right)}^{3.19257}\right]$$

Using the DRX volume fraction curves, it was possible to obtain the *ε*_0.5_-value. Taking the natural logarithm of Equation (22), Equation (27) can be obtained.

(27) $$\ln\varepsilon_{0.5}=\ln A_{5}+m_{5}\ln\dot{\varepsilon }+Q_{5}/RT$$

From the above equation, it can be seen that $\ln\varepsilon_{0.5}$ and $\ln\dot{\varepsilon }$ show an obvious linear relationship when *T* is certain, and the average the value of *m*_5_ is 0.064. Similarly, lnε_0.5_ and *T*^−1^ is a linear relationship at a given $\dot{\varepsilon }$, and the slope is calculated to be *Q*_5_/R = 7.1192 with an intercept of −6.8866; therefore, *Q*_5_ is 59189.028 J/mol, and *A*_5_ is 6.77 × 10^−4^.

Substituting the above values into Equation (27) yields Equation (28).

(28) $$\varepsilon_{0.5}=6.77054\times 10^{-4}{\dot{\varepsilon }}^{0.17852}\exp\left(59189.028/RT\right)$$

In summary, the DRX kinetics of the novel GH4720Li superalloy is modeled, as shown in Equation (29):

(29) $$\left\{\begin{array}{l} X_{drx}=1-\exp\left[-0.5308{\left(\frac{\varepsilon -0.72551\varepsilon_{p}}{\varepsilon_{0.5}}\right)}^{3.19257}\right]\\ \varepsilon_{0.5}=6.77054\times 10^{-4}{\dot{\varepsilon }}^{0.17852}\exp(59189.028/RT) \end{array}\right.$$

**Figure 10:**
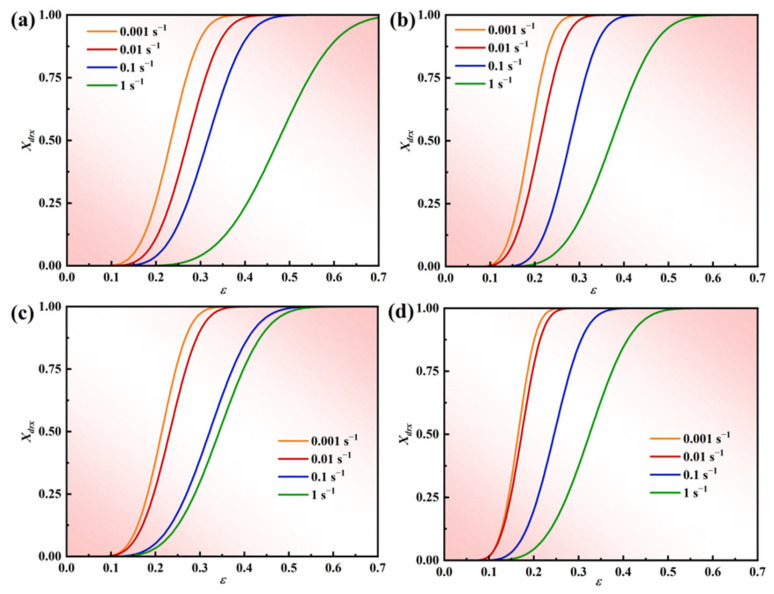
The correlation between *X_drx_* and true strain for the novel GH4720Li superalloy under various deformation conditions: (**a**) 950 °C, (**b**) 1000 °C, (**c**) 1050 °C, and (**d**) 1100 °C.

Figure 10 depicts the correlation between *X_drx_* and the true strain for the novel GH4720Li superalloy under various deformation conditions. A comparison of the *X_drx_* curves reveals that higher temperatures and lower strain rates increase the material’s susceptibility to DRX. This is due to slower vacancy atom diffusion at lower temperatures, which impedes cross-slip and climb processes. Additionally, higher strain rates intensify dislocation and grain boundary interactions, promoting dislocation movement along new slip surfaces, both hindering DRX nucleation. Consequently, DRX is more easily created at higher deformation temperatures and lower strain rates.

#### 3.3.3. Influence of Deformation Amount on the DRX of Alloys

Significant changes occur in the grain size, morphology, and grain boundaries of the alloy during hot deformation. The DRX is significantly influenced by the deformation parameters. Therefore, investigating the microstructure evolution of the novel GH4720Li superalloy under different reductions is imperative.

**Figure 11:**
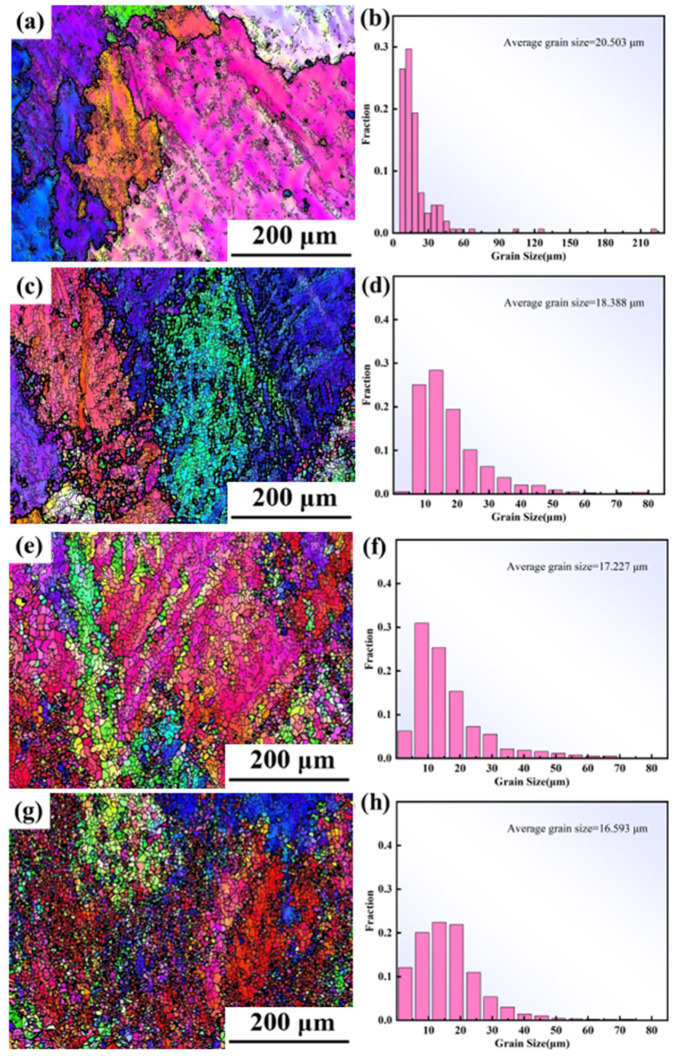
The IPF and GOD of the novel GH4720Li alloy compressed at 950 °C/1 s^−1^ under true strains of (**a**,**e**) 0.2, (**b**,**f**) 0.3, (**c**,**g**) 0.4, and (**d**,**h**) 0.5.

**Figure 12:**
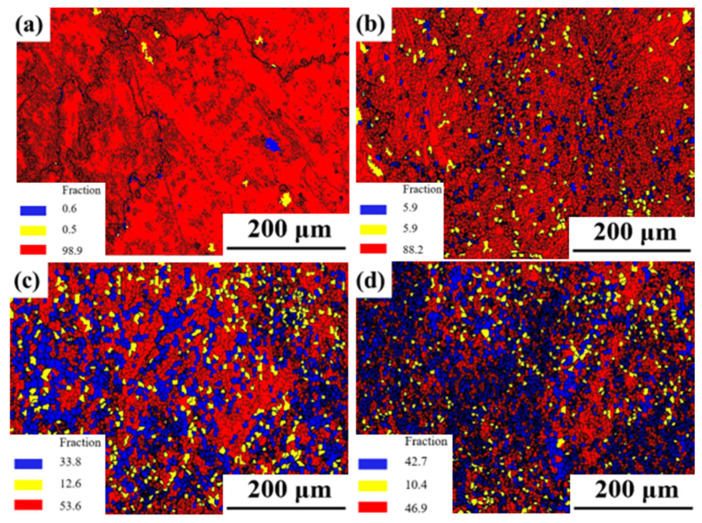
Grain types of the novel GH4720Li alloy compressed at 950 °C and 1 s^−1^ under different true strains (**a**) 0.2, (**b**) 0.3, (**c**) 0.4, and (**d**) 0.5.

Figure 11 illustrates the inverse pole figure (IPF) and grain orientation distribution (GOD) of the novel GH4720Li alloy under various deformation levels at 950 °C/1 s^−1^. Analysis of Figure 11a indicates that DRX initiates at a true strain of 0.2, coexisting with numerous elongated initial grains. Simultaneously, irregular deformation manifests at certain grain boundaries, accompanied by DRX in their vicinity, albeit with a minimal number of grains undergoing such transformation. Currently, grain sizes predominantly range between 30 μm and 240 μm, signifying their status as original grains following compression deformation. As the true strain rises to 0.3, numerous small isometric crystals emerge surrounding the elongated grains, creating a necklace-like configuration with the original grains (see Figure 11b). Most grains are currently below 30 μm in size, indicative of the further compression and deformation of the initial grains, with smaller grains primarily constituting DRXed ones. Subsequent to a further increase in deformation to 0.4 and 0.5, elongated grain size notably diminishes, accompanied by a substantial increase in the number of DRXed grains (Figure 11c,d). Presently, grain size primarily concentrates below 30 μm, with the majority comprising DRXed grains. Concurrently, an examination of the rheological stress curves (Figure 2) at 950 °C, a strain rate of 1 s^−1^, and a deformation level of 70% reveals an initial increase followed by a subsequent decrease. Using EBSD statistics, the DRX percentages shown in Figure 12a–d are 0.6%, 5.9%, 33.8%, and 42.7%, respectively. Based on the curve shown in Figure 10a and recrystallization experiments of alloys with different strains, a comparative analysis of the experimental and theoretical results proves that the calculated DRX model is feasible.

Figure 12 shows the grain types of the novel GH4720Li alloy compressed at 950 °C/1 s^−1^ under different true strains. In this figure, red signifies deformed grains, yellow represents the substructure, and blue indicates recrystallized grains. Initially, at 950 °C and low deformation levels, the alloy predominantly consists of red deformed grains, with a minimal presence of a yellow substructure and blue recrystallized grains. The proportion of deformed grains is notably high at 98.9%, while the substructured and DRXed grains collectively account for only 1.1%. However, as deformation increases, the proportion of DRXed grain gradually rises. At a deformation level of 0.3, the percentage of DRXed grain increases to 5.9%, accompanied by a decrease in the proportion of deformed grain to 88.2%. At a deformation level of 0.4, the percentage of DRXed grain is 33.8%, accompanied by a sharp decrease in the proportion of deformed grain from 88.2% to 53.6%. This reduction occurs because sufficient DRX takes place when dislocations accumulate to a critical value during the deformation process, thereby promoting DRX nucleation and grain growth. As a result, most of the deformed microstructure transforms into DRX grains due to DRX behavior. However, upon reaching a deformation level of 0.5, there is an increase in DRXed grain. The main reason for the reduced increase in recrystallization is that some of the dislocations within the alloy have been consumed.

**Figure 13:**
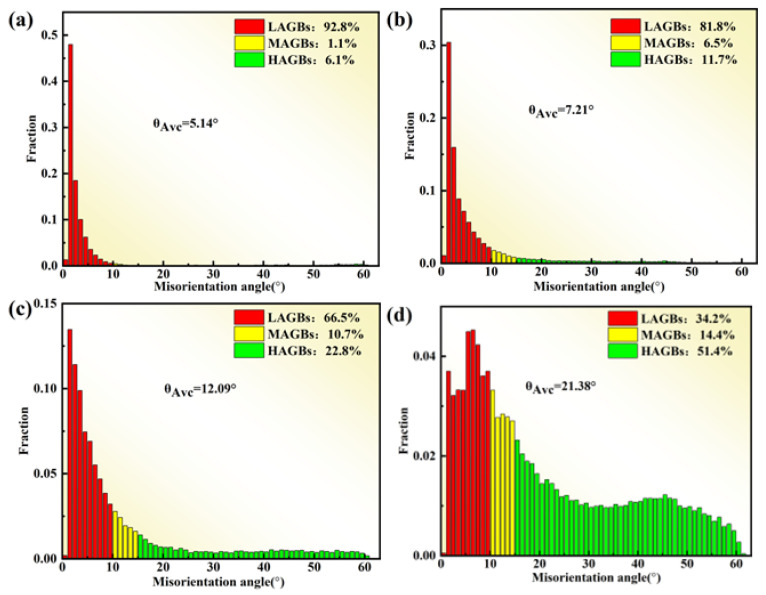
DRXed grain of the novel GH4720Li alloy compressed at 950 °C and 1 s^−1^ under different true strains: (**a**) 0.2, (**b**) 0.3, (**c**) 0.4, and (**d**) 0.5.

Figure 13 shows the variation of different GB orientations of the novel GH4720Li alloy compressed at 950 °C/1 s^−1^. The increase in the proportion of LAGBs (LAGBs, <10°) when the amount of deformation is 0.2 and 0.3 indicates that as deformation increases, more dislocations accumulate and entangle in the alloy, resulting in an increase in the proportion of LAGBs. In general, continuous dynamic recrystallization (CDRX) occurs within the deformed grains and is associated with subcrystalline forms and dislocations, while mid-angle grain boundaries (MAGBs, 10°~15°) are considered to be necessary for the occurrence of CDRX. Meanwhile, the MAGBs show an increasing trend, which indicates that CDRX behavior occurs during hot deformation. When the amount of deformation increases to 0.4 and 0.5, the proportion of LAGBs decreases while the proportion of high-angle grain boundaries (HAGBs, >15°) increases, indicating that when the amount of deformation reaches 0.4 and 0.5, the over-absorbed dislocations are converted to HAGBs; then, dislocation-free recrystallized grains are formed, and the proportion of dynamic recrystallization increases, while the proportion of MAGBs continues to increase with the amount of deformation. MAGBs act as a transition zone from LAGBs to HAGBs; therefore, they can be used as a marker of whether the CDRX mechanism is active or not. However, the proportion of MAGBs in the alloys is always small, which suggests that the CDRX mechanism is only an auxiliary dynamic recrystallization mechanism under different deformation amounts.

**Figure 14:**
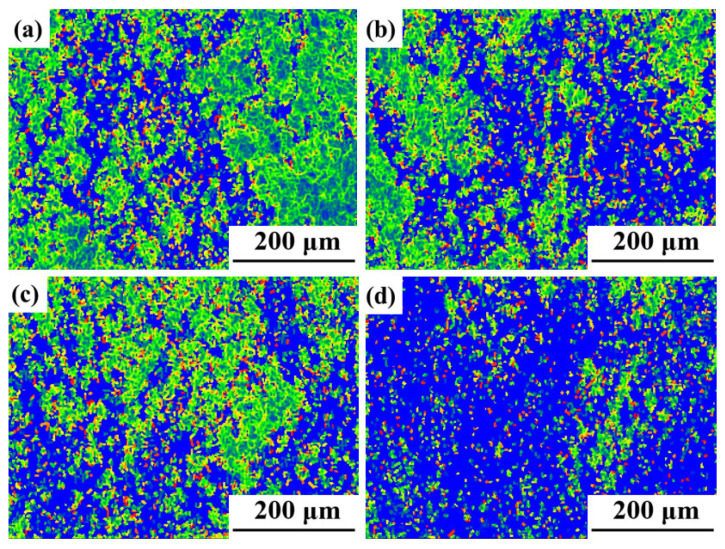
GROD of the novel GH4720Li alloy at 950 °C and 1 s^−1^ under different true strains: (**a**) 0.2, (**b**) 0.3, (**c**) 0.4, and (**d**) 0.5.

Figure 14 illustrates the grain reference point orientation deviation (GROD) at 950 °C/1 s^−1^. GROD plots depict orientation deviations from minimum to maximum, indicated in a gradient from blue to red. These deviations arise from orientation undulations due to dislocation accumulation, implying that regions with higher GROD values exhibit similarly elevated dislocation densities. Observing the figure, during hot deformation, dislocation storage in initially deformed grains is substantial, while DRXed grains accumulate very few dislocations. This suggests that DRX promptly eliminates dislocations, leading to a reduction in dislocation density. New DRXed grains can then readily accumulate enough dislocations to form within the deformed grains. When the dislocation density in a particular region reaches a critical value, the material initiates DRX. Moreover, dislocation accumulation near grain boundaries markedly surpasses that within boundaries, indicating that new DRXed grains primarily form along deformed grain boundaries. This delineates a dynamic process where dislocation accumulation and subsequent DRX play pivotal roles in microstructural evolution during hot deformation.

## 4. Conclusions

In this study, the constitutive modeling and DRX mechanism were established based on the stress–strain curve of the novel GH4720Li alloy. The results are as follows.

1. During the hot compression process of the novel GH4720Li alloy, dislocations proliferate and accumulate with increasing strain. When the strain reaches the critical value, DRX occurs. The critical strain is influenced by the deformation temperature, strain rate, and microstructure, reflecting the difficulty of DRX. According to the Sellars model, the critical strain model for the novel GH4720Li alloy during hot deformation is obtained:

$$\left\{\begin{array}{c} \varepsilon_{c}=0.69722\varepsilon_{p}\\ \varepsilon_{p}=0.07127{\dot{\varepsilon }}^{0.07016}\exp(35562.533/RT) \end{array}\right.$$

2. The DRX volume fraction reflects the extent of the DRX process, determining the final mechanical properties. It can be calculated using the JMAK model for the GH4720Li alloy as follows:

$$\left\{\begin{array}{l} X_{drx}=1-\exp\left[-0.5308{\left(\frac{\varepsilon -0.72551\varepsilon_{p}}{\varepsilon_{0.5}}\right)}^{3.19257}\right]\\ \varepsilon_{0.5}=6.77054\times 10^{-4}{\dot{\varepsilon }}^{0.17852}\exp(59189.028/RT) \end{array}\right.$$

3. Contrastive analysis of the experimental results and the theoretical results proved that the DRX model of a novel GH4720Li superalloy established through calculation is feasible. During the compression of the novel GH4720Li superalloy, DRX initiates when the dislocation density in a specific region surpasses a critical threshold. Concurrently, dislocation accumulation near the grain boundaries exceeds that within the grains themselves, highlighting that newly formed DRXed grains primarily emerge along the deformed grain boundaries.

## Figures and Tables

**Table 1:** Chemical composition of superalloy (wt./%).

C	B	Co	Cr	Mo	Al	Ti	W	Y	Ni
0.02	0.012	14.11	16.0	3.01	2.50	5.03	1.25	0.34	Bal.

## Data Availability

The original contributions presented in the study are included in the article, further inquiries can be directed to the corresponding author.
